# Supplementary material for: Longer chronic cannabis use in humans is associated with impaired implicit motor learning and supranormal resting state cortical activity
Source: PLoS One. 2026 Jan 7;21(1):e0338082. doi: 10.1371/journal.pone.0338082 (PMC12779070; doi:10.1371/journal.pone.0338082)
Supplement: S1 Table — (DOCX) [file pone.0338082.s001.docx]

# Supporting Information

**Table S1. Partial correlations (controlled for alcohol use) between variables related to cannabis use and cognitive-emotional assessments, the serial reaction time (SRT) task, the Corsi block-tapping task, and spectral power in the cannabis group.**

|  | Lifetime cannabis use | Years of cannabis use | Past month cannabis use | Age at onset of cannabis use | CUDIT-R | MPS | MCQ-SF (Session 2) | MCQ-SF (Session 3) | |
| --- | --- | --- | --- | --- | --- | --- | --- | --- | --- |
| Cognitive-Emotional Assessments | | | | | | | | |  |
| DASS-21 |  |  |  |  |  |  |  |  | |
| Depression | 0.15 | -0.017 | 0.075 | -0.13 | 0.20 | 0.26 | - | - | |
| Anxiety | 0.13 | 0.14 | 0.14 | -0.27 | 0.15 | 0.30* | - | - | |
| Stress | 0.14 | -0.21 | -0.11 | -0.059 | -0.084 | 0.26 | - | - | |
| CFQ | -0.022 | -0.23 | -0.16 | -0.20 | -0.003 | 0.30* | - | - | |
| MoCA | -0.17 | -0.086 | 0.045 | 0.37* | -0.011 | -0.067 | - | - | |
|  |  |  |  |  |  |  |  |  | |
| Serial Reaction Time Task | | | | | | | | |  |
| Index of motor learning | -0.081 | -0.55* | -0.15 | 0.33 | -0.022 | -0.13 | -0.15 | - | |
|  |  |  |  |  |  |  |  |  | |
| Corsi Block-Tapping Task | | | | | | | | | |
| Forward span | -0.14 | -0.19 | 0.013 | 0.070 | -0.13 | -0.16 | -0.18 | - | |
| Backward span | -0.093 | -0.046 | -0.021 | 0.12 | -0.092 | -0.15 | -0.37* | - | |
|  |  |  |  |  |  |  |  |  | |
| EEG |  |  |  |  |  |  |  |  | |
| Delta |  |  |  |  |  |  |  |  | |
| Frontal | 0.17 | 0.15 | -0.020 | 0.14 | 0.084 | 0.082 | - | -0.32 | |
| Central | 0.074 | 0.14 | -0.18 | 0.27 | 0.038 | 0.032 | - | -0.29 | |
| Parietal | -0.012 | 0.13 | -0.16 | 0.19 | -0.002 | 0.010 | - | -0.37* | |
| Occipital | -0.065 | 0.033 | -0.23 | 0.19 | -0.21 | -0.23 | - | -0.38* | |
| Theta |  |  |  |  |  |  |  |  | |
| Frontal | -0.21 | -0.18 | -0.040 | -0.021 | -0.36 | -0.19 | - | 0.21 | |
| Central | -0.16 | -0.15 | -0.017 | -0.070 | -0.35 | -0.18 | - | 0.19 | |
| Parietal | -0.17 | -0.19 | -0.049 | -0.069 | -0.30 | -0.11 | - | 0.21 | |
| Occipital | -0.19 | -0.23 | -0.059 | 0.009 | -0.33 | -0.094 | - | 0.22 | |
| Alpha |  |  |  |  |  |  |  |  | |
| Frontal | -0.17 | -0.069 | -0.079 | -0.13 | 0.018 | -0.061 | - | 0.17 | |
| Central | -0.068 | -0.040 | 0.054 | -0.13 | -0.018 | -0.092 | - | 0.16 | |
| Parietal | 0.061 | -0.053 | 0.160 | -0.092 | 0.096 | -0.024 | - | 0.27 | |
| Occipital | 0.085 | 0.077 | 0.16 | -0.15 | 0.30 | 0.21 | - | 0.19 | |
| Beta |  |  |  |  |  |  |  |  | |
| Frontal | 0.062 | -0.12 | 0.27 | -0.38 | 0.11 | 0.081 | - | 0.49* | |
| Central | 0.12 | -0.10 | 0.26 | -0.44* | 0.23 | 0.24 | - | 0.34 | |
| Parietal | 0.15 | -0.021 | 0.20 | -0.37 | 0.20 | 0.22 | - | 0.30 | |
| Occipital | 0.21 | -0.042 | 0.26 | -0.22 | 0.22 | 0.26 | - | 0.33 | |
| Gamma |  |  |  |  |  |  |  |  | |
| Frontal | 0.19 | -0.11 | 0.62* | -0.20 | 0.20 | 0.21 | - | 0.45* | |
| Central | 0.015 | -0.14 | 0.43* | -0.24 | 0.27 | 0.16 | - | 0.16 | |
| Parietal | 0.12 | 0.074 | 0.26 | -0.24 | 0.30 | 0.19 | - | 0.018 | |
| Occipital | 0.17 | -0.076 | 0.48* | -0.19 | 0.057 | 0.11 | - | 0.51* | |

Abbreviations: CUDIT-R, Cannabis Use Disorder Identification Test-Revised; MPS, Marijuana Problem Scale; MCQ-SF, Marijuana Craving Questionnaire-Short Form, DASS-21, Depression, Anxiety, and Stress Scale – 21 Items; CFQ, Cognitive Failures Questionnaire; MoCA, Montreal Cognitive Assessment.

*Indicates *p* < 0.05
